# Successful pregnancy of a Maine Coon queen despite feline mammary fibroadenomatous hyperplasia recurrence after treatment with aglepristone

**DOI:** 10.17221/51/2024-VETMED

**Published:** 2025-01-15

**Authors:** Piotr Socha, Pawel Mossakowski

**Affiliations:** ^1^Department of Animal Reproduction with Clinic, Faculty of Veterinary Medicine, University of Warmia and Mazury in Olsztyn, Olsztyn, Poland; ^2^Private Veterinary Practice “Zwierzyniec”, Gdansk, Poland

**Keywords:** antiprogestagen, cat, FMFH

## Abstract

One of the disorders of the mammary gland in the queen is feline mammary fibroadenomatous hyperplasia (FMFH), caused by an increasing concentration of progesterone (P4) and some other local growth factors. It occurs mostly during puberty after the heat characterised by spontaneous or provoked ovulation, as a result of exogenous progesterone intake and sometimes during pregnancy. To diagnose a 14-month-old intact Maine Coon queen with extensive mammary gland hyperplasia, a clinical examination, analyses of the progesterone (P4) concentrations and ultrasound examination were performed. Feline mammary fibroadenomatous hyperplasia associated with a high P4 concentration after spontaneous ovulation was confirmed. After 24 days of therapy with a progesterone antagonist, aglepristone, the symptoms of FMFH resolved. After the next eight weeks, the queen was mated after the owner’s decision. In the third week of pregnancy, a relapse was detected (mammary gland enlargement, pain, discomfort). At the same time, no abnormalities in the uterus or embryos were detected via ultrasound. The P4 concentrations were under regular control. For the next two weeks, only conservative treatment with NSAIDs was used. The queen spontaneously delivered six kittens without any difficulties or perinatal complications 67 days after the first mating. The cat previously treated with aglepristone for FMFH was successfully bred, but FMFH symptoms returned when progesterone concentrations increased during pregnancy.

Feline mammary fibroadenomatous hyperplasia (FMFH) is a condition characterised by stromal and epithelial proliferation of mammary glands in cats (Torrigiani et al. 2022). Fibroadenoma is a hormone-dependent disorder resulting from a high concentration of progesterone (P4) and coexistence of several other local growth factors. As progesterone induces growth hormone (GH) release, the pathogenesis of FMFH might be related to the synergism between progesterone, oestrogens (E), GH and insulin-like growth factor (IGF-1) in the mammary tissue (Martin de las Mulas et al. 2000; Ordas et al. 2004). This leads to an overgrowth of ductal epithelium and periductal connective tissue, significantly enlarging single or multiple mammary glands at the beginning without any signs of inflammation. High P4 concentrations can occur during pregnancy, pseudopregnancy (nonpregnant luteal phase) or all progesterone therapies (Verhage et al. 1976; Shille and Stabenfeldt 1979; Zschockelt et al. 2014). In a study describing 14 cats with fibroadenomatosis, 50% of the cases were the result of hormone therapy (six cats received medroxyprogesterone acetate, and one received proligestone), whereas seven were not iatrogenic (Jurka and Max 2009). There are also case reports of FMFH observed in tom cats as a result of functional ovarian tissue or due to treatment with megestrol acetate ordered because of suspected eosinophil–granuloma complexes (MacDougall 2003; Mayayo et al. 2018). Fibroadenoma can be seen on ultrasound as a solid mass with fluid accumulation or as a parenchymal intraductal pattern with spaces containing fluid (Payan-Carreira 2013). Chronic mammary gland hyperplasia can progress to mastitis and abscessation of the mammary glands (Burstyn 2010). The recommended conservative treatment of FMFH is based on the the progesterone antagonist aglepristone (Alizin) in combination with antibiotics and anti-inflammatory drugs (Marino et al. 2021). FMFH management differs in the dose of Alizin administered subcutaneously, ranging from 10 mg/kg to 20 mg/kg per day, and in intervals between doses and duration of treatment (Gorlinger et al. 2002; Jurka and Max 2009; Marino et al. 2021). Remarkably, the most effective method to prevent FMFH is to spay and exclude the queen from breeding. Nevertheless, it was reported that queens treated for FMFH can still be mated successfully without relapse of mammary gland hyperplasia (Jurka and Max 2009).

## Case presentation

**Figure 1:**
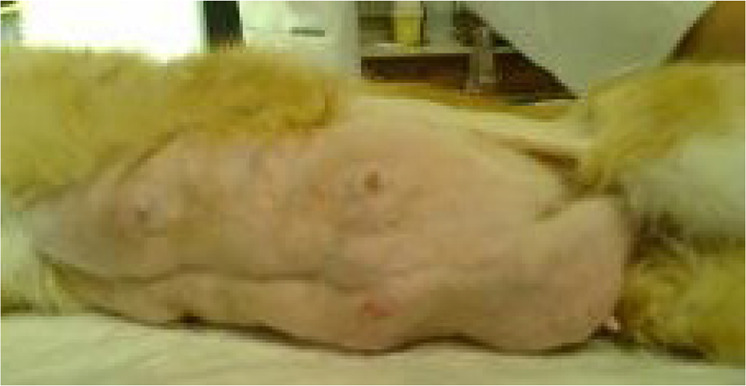
Clinical abnormal enlargement of the mammary gland in examined cat

**Figure 2:**
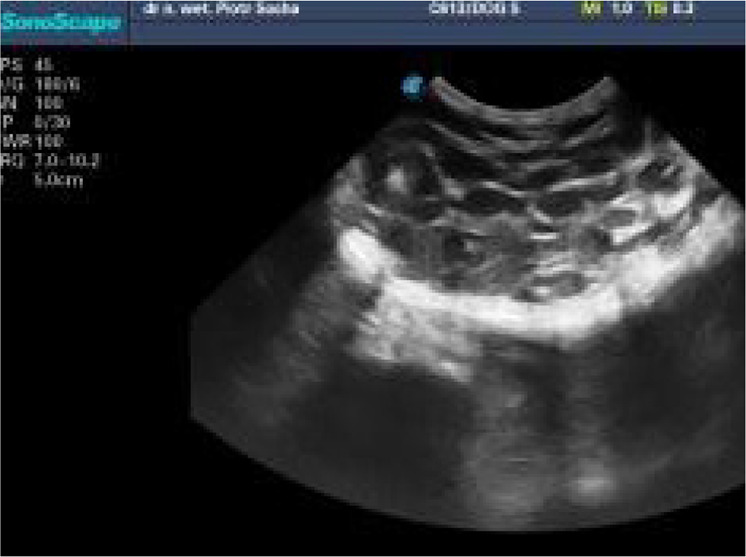
The US view of abnormal mass of mammary gland during fibroadenomatosis

A 14-month-old intact Maine Coon queen weighing 6.8 kg was examined because of the owners’ concerns regarding an abnormal mass on the abdomen. In the treatment history, the queen did not receive any exogenous progesterone. In the clinical examination, general parameters such as body temperature, mucous membrane colour, heart rate and respiratory rate were within physiological values. A remarkable enlargement of the mammary gland was confirmed (Figure 1). No signs of inflammation or skin ulceration were seen, excluding mastitis from the differential diagnosis. In the ultrasound examination, pregnancy was excluded, but a solid mass of mammary gland with fluid accumulation and a parenchymal intraductal pattern with spaces containing fluid were confirmed (Figure 2). Based on the clinical results, ultrasonography and progesterone analyses, FMFH was confirmed.

The cat was treated with 15 mg/kg aglepristone (Alizine; Virbac, Carros Cedex, France) administered subcutaneously. Alizine was administered according to the manufacturer’s recommendations: on day 1, 48 h after the starting dose, and then within five-day intervals until symptoms resolved. Alizine therapy was fully effective after the second dose, leading to complete remission after twenty-four days since the start of treatment. During eight weeks of follow-up, no relapse was revealed. This was one of the reasons why the owner himself decided to mate the queen.

The first ultrasound examination performed 21 days after mating confirmed proper gestation. At the next visit on the 25^th^ day after mating, a relapse consisting of enlargement of the mammary glands was observed. Despite the information concerning the cat’s life and health, the owner decided to maintain her pregnancy.

During the next days, blood samples were collected for immunofluorescence analysis of the P4 concentration. Tests for P4 concentrations were performed consecutively on days 25, 27, and 34 of pregnancy, and the results obtained were 32.6 ng/ml, 39.5 ng/ml, and 47.3 ng/ml, respectively. Between days 43 and 57 of pregnancy, the P4 concentration gradually decreased from 31.4 ng/ml to 8 ng/ml. From day 25 until 42 of pregnancy, only single-drug therapy with a nonsteroid anti-inflammatory drug, Tolfedine (Acidum tolfenamicum) 6 mg in tab. (Vetoquinol S.A.; Magny-Vernois, Lure Cedex, France). Dosage: 4 mg/kg b.w. once per day for 3 days, followed by 4 days of rest and 17 days of therapy. Physiologically decreasing P4 concentrations near the end of pregnancy resulted in maintaining the current rate of the mammary gland at a fixed level, with no signs of ulceration or necrosis. From day 45 of pregnancy, the abnormal growth of the mammary gland has slowed down, and this has not affected its natural development associated with preparation for physiological lactation after delivery. The queen spontaneously delivered six kittens without any difficulties or perinatal complications 67 days after the first mating. A complication of mastitis appeared seven days postpartum without influencing lactation and was treated with amoxicillin and clavulanic acid for 7 days and tolfenamic acid for 4 days. All the procedures described and administered in this clinical case are summarised and presented in Table 1.

**Table 1:** Description and procedures used for the clinical case history of a pregnant queen with FMFH previously treated with aglepristone

Day after mating	Description/conducted procedures
21	ultrasound (US) examination – confirmation of gestation
25	first symptoms of feline mammary fibroadenomatous hyperplasia (FMFH) progesterone (P_4_) concentration in blood – 32.6 ng/ml initiation of seventeen-days therapy with Tolfedine 6 mg in tablet form
27	FMFH US examination – physiological pregnancy P_4_ concentration – 39.5 ng/ml
34	FMFH P_4_ concentration – 47.3 ng/ml
43	FMFH P_4_ concentration – 31.4 ng/ml
45	symptoms of FMFH slowed down
49	US examination – physiological pregnancy P_4_ concentration – 22.6 ng/ml
51	P_4_ concentration – 13.4 ng/ml
57	P_4_ concentration – 8 ng/ml
67	natural delivery

## DISCUSSION AND CONCLUSIONS

This case showed that despite previous treatment of fibroadenoma with aglepristone therapy, a queen can still develop FMFH during the next period of increased P4 concentrations. One cycle of aglepristone therapy affects only the current concentration of the described hormone and its receptors, while the effect does not persist over time. Some cases of FMFH may require surgical treatment, such as partial or total mastectomy (Payan-Carreira 2013). Surgical intervention in mammary glands or gonads (ovariohysterectomy or ovariectomy) may lead to a loss of breeding value for the queen. Such invasive treatments are not the first choice for breeder-owned cats, but conservative treatment seems to be more cost-effective from an economic point of view. To the best of our knowledge, this is the first full clinical report of successful delivery in a female cat that was mated after developing FMFH in a previous oestrus cycle. The queen was able to maintain pregnancy and give birth to healthy kittens while developing FMFH again.

Tolfedine is the only NSAID approved in Poland for use in pregnant and lactating cats. When planning the conservative therapy with tolfenamic acid in this case, all doubts regarding the use of NSAIDs in a pregnant cat were taken into account. It is known that prolonged treatment with NSAIDs can result in gastrointestinal effects (such as loss of appetite, vomiting, diarrhoea, blood in the faeces), kidney and liver injury, and also impaired coagulation, so the duration of the treatment given has been kept to a minimum (Taylor et al. 2024). In addition, the kittens need to be monitored, as being born clinically healthy is no guarantee of continued good health. In human medicine, serious adverse effects on the foetus have been described when NSAIDs were administered during pregnancy. There were reports of congenital malformations, antepartum haemorrhage, oligohydramnios, and increased risk of miscarriages (Nielsen 2001; Choi et al. 2023). Unfortunately, safety data specific to cats are weak. Although the premise of this clinical case was not to monitor the health and development of the kittens in the days following birth, the authors were informed that all kittens developed normally during the first 14 days after birth.

FMFH is a fairly common condition in breeding cats and results in significant economic losses to owners. The results obtained refer to only one patient, but the fact that healthy kittens were delivered indicates the necessity to carry out further studies with a larger group of individuals, which should include a homogenous population, as much as possible, in terms of age and breed. The authors believe that the positive final results of this report indicate that further research is necessary. The results could be applied to new approaches for cat breeding.

In conclusion, we confirmed that fibroadenomatosis can be effectively treated with aglepristone and cats after this therapy can be successfully bred. However, it should be emphasised, that there is a risk of FMFH recurrence if the blood concentrations of P4 increase during pregnancy. Moreover, even if symptoms of fibroadenomatosis appear in mid-pregnancy, there is a chance of successful pregnancy, natural delivery and normal lactation in cats.
